# The Influence of Long Term Hydrochlorothiazide Administration on the Relationship between Renin-Angiotensin-Aldosterone System Activity and Plasma Glucose in Patients with Hypertension

**DOI:** 10.1155/2013/434618

**Published:** 2013-11-19

**Authors:** Xu Xiao, Hong-jun Du, Wei-jian Hu, Peter X. Shaw

**Affiliations:** ^1^Department of Emergency Medicine, The Sichuan Provincial People's Hospital, Chengdu 610072, China; ^2^Department of Ophthalmology, University of California, San Diego, 9500 Gilman Drive, La Jolla, CA 92093, USA

## Abstract

*Objective*. To observe the relationship between changes in renin-angiotensin-aldosterone system (RAAS) activity and blood plasma glucose after administration of hydrochlorothiazide (HCTZ) for one year in patients with hypertension. *Methods*. 108 hypertensive patients were given 12.5 mg HCTZ per day for one year. RAAS activity, plasma glucose levels, and other biochemical parameters, as well as plasma oxidized low density lipoprotein (oxLDL) levels, were measured and analyzed at baseline, six weeks, and one year after treatment. *Results*. After one year of treatment, the reduction in plasma glucose observed between the elevated plasma renin activity (PRA) group (−0.26 ± 0.26 mmol/L) and the nonelevated PRA group (−1.36 ± 0.23 mmol/L) was statistically significant (*P* < 0.05). The decrease of plasma glucose in the elevated Ang II group (−0.17 ± 0.18 mmol/L) compared to the nonelevated Ang II group (−1.07 ± 0.21 mmol/L) was statistically significant (*P* < 0.05). The proportion of patients with elevated plasma glucose in the elevated Ang II group (40.5%) was significantly higher than those in the nonelevated Ang II group (16.3%) (*P* < 0.05). The relative oxLDL level was not affected by the treatment. *Conclusions*. Changes in RAAS activity were correlated with changes in plasma glucose levels after one year of HCTZ therapy.

## 1. Introduction

The renin-angiotensin-aldosterone system (RAAS) is composed of a series of hormones and corresponding enzymes. By controlling the blood volume and peripheral resistance, RAAS helps maintain the balance between human blood pressure, water and electrolytes, and thus homeostasis. Currently, the levels of plasma renin activity (PRA), angiotensin II (Ang II), and aldosterone (ALD) have become the key indicators for diagnosis, treatment, and clinical research about both primary and secondary types of hypertension. Research has demonstrated that RAAS activation not only was an important mechanism for the development of hypertension, but also could modulate insulin resistance [1]. Patients with hypertension usually exhibit insulin resistance and the risk of diabetes is elevated compared to nonhypertensive patients [2]. Consequently, as first-line antihypertensive medications, diuretics may influence RAAS [3]. However, little research has examined the relationship between changes in RAAS and changes in plasma glucose level. Thus, we examined changes in RAAS activity and plasma glucose in primary hypertensive patients taking HCTZ for one year, with the hope of providing new insight into the study of hypertension and its treatment.

## 2. Materials and Methods

### 2.1. Patient Enrollment

From November 2007 to October 2008, 108 patients diagnosed with primary hypertension in Liangshan First People's Hospital in China were recruited. Inclusion criteria are (1) older than 18 years, either sex; (2) Han ethnicity; (3) diagnosis of hypertension based on World Health Organization and the International Union of Hypertension (WHO/ISH) diagnosis and grading standards issued in 1999: mild to moderate hypertension refers to those who had 3 consecutive systolic blood pressures of 140 to 179 mmHg (1 mmHg = 133.32 Pa) and/or diastolic blood pressures of 90 to 109 mmHg in the sitting position measured on different days. Exclusion criteria are (1) secondary hypertension; (2) severe renal and hepatic dysfunction; (3) severe valvular heart disease, cardiomyopathy, and unstable angina or undergoing coronary artery bypass surgery in 6 months; (4) gout or diabetes mellitus; (5) never taken antihypertensive medications. The ethics committee at each participating center approved the study. All eligible subjects participated voluntarily and written informed consent was obtained from all patients.

### 2.2. Medication Administration

All 108 patients were given 12.5 mg HCTZ (Southwest Pharmaceutical Co., Ltd., batch number: 0707002 Chongqing, China) by mouth once daily for one year and the follow-up interval was one month. Dosage was increased to 25 mg in patients with inadequate blood pressure control. A variety of biochemical indicators were tested at baseline, six weeks and one year after beginning treatment. 

### 2.3. Clinical Observations 

#### 2.3.1. General Data Collection

General information that was collected or calculated included gender, age, height, weight, body mass index (BMI), and previous medical history. Standard program was used to calculate height and body mass index.

#### 2.3.2. Blood Pressure Measurement

Blood pressure was measured while the patient was seated after at least 5 min and had not smoked for 15 min before each measurement. Calibrated mercury sphygmomanometer was used to measure blood pressure. Two weeks before drug administration, three measurements were taken and the mean pressure levels were considered as the baseline pressure. All measurements were standardized: the same time (8:00 a.m. to 9:00 a.m.), arm, sphygmomanometers, and doctor.

#### 2.3.3. Blood Sample Collection and Biochemical Indicators Measurement

Patients avoided vigorous activity and maintained normal eating habits for 3 days prior to blood draws. Blood samples were drawn at 8 am after overnight fasting to measure laboratory biochemical parameters, including blood urea nitrogen (BUN), creatinine (Cr), plasma glucose (GLU), blood potassium (K^+^), triglyceride (TG), total cholesterol (CHO), high density lipoprotein cholesterol (HDL-C), low density lipoprotein cholesterol (LDL-C), and very low density lipoprotein cholesterol (VLDL-C). The measurement was performed in the biochemistry laboratory of Sichuan Provincial People's Hospital using automated biochemical analyzer (OLYMPUS AU5400). The blood drawings were conducted at the time of enrollment for baseline, six weeks, and one year after beginning HCTZ treatment.

#### 2.3.4. ELISA

Sandwich enzyme-linked immunosorbent assay (ELISA) was used to measure the plasma level of oxLDL as previously described [4]. Goat anti-human apoB (Sigma, St. Louis, MO, USA) was coated on a 96-well microtiter plate as the capturing antigen. A 1 : 100 dilution of plasma was added to the plate and followed by monoclonal anti-oxPL antibody TEPC-15 (Sigma, St. Louis, MO, USA). The amount of bound oxLDL was detected with biotinylated anti-mouse IgA followed by neutral avidin-alkaline phosphatase (AP). The light emission substrate Lumi-Phos 530 was added and the chemiluminescence was measured by GloMax Luminometer (Promega) and expressed as relative light units (RLU).

#### 2.3.5. Detection of RAAS Activity

For the RAAS activity determinations, venous blood was taken from a vein in the antecubital fossa in the presence of the anticoagulant heparin. Serum angiotensin converting enzyme (ACE) levels and ALD levels were measured in the dual-points ending-point determination method. Diagnostic kits were purchased from Beijing Shizhen Zhongtuo Biotechnology Co., Ltd., or provided by Northern Biotechnology Research Institute. PRA and Ang II levels were measured by radioimmunoassay in venous blood after adding enzyme inhibitors (included in the kit). Diagnostic kits were also provided by Northern Biotechnology Research Institute. All measurements were performed according to the manufacturers' protocols.

### 2.4. Statistical Analysis

SPSS 13.0 software was used to establish and analyze the database. Numeric variable data is expressed as mean ± standard deviation. Paired *t*-test was used to compare the values at baseline and those after one year of treatment. Levels of plasma GLU and blood K^+^ in different RAAS groups were compared with Two-Sample Comparison *t*-test. Chi-square test and multivariate linear regression analysis were used for multifactorial variables. *P* < 0.05 was considered as statistically significant.

## 3. Results

### 3.1. Patients

All 108 patients (68 males, 40 females) completed this study during their one-year drug administration and followups. The average age was 57.0 ± 8.1 years, and mean BMI was 27.2 kg/m^2^ ± 3.4 kg/m^2^. After treatment with HCTZ for one year, GLU, CHO, LDL-C, PRA, and Ang II levels in all patients were significantly decreased and HDL-C levels were increased significantly. However, there were no significant changes in other indexes (Table 1).

### 3.2. Relationship between GLU Concentration and RAAS Activity

According to the changes in RAAS activity after one year of medication, patients were divided into elevated and nonelevated RAAS groups. Glucose concentrations and changes were compared between these two groups (Table 2).

There were no statistically significant differences (*P* > 0.05) between the GLU concentrations of patients with elevated PRA and Ang II levels and those in the nonelevated patient groups (Table 2), despite the increasing tendency. However, the GLU concentration reductions in patients with elevated PRA and Ang II levels were statistically significantly lower (*P* < 0.05) than those in the nonelevated patient groups. The reductions of GLU concentration in patients with elevated ACE and ALD concentration were lower than those in nonelevated patients; however, the differences were not statistically significant (*P* > 0.05).

### 3.3. Relationship between Changes in GLU and RAAS Activity after Medication

According to changes in RAAS and GLU levels after 1 year of medication, patients were divided into either elevated or nonelevated groups. The proportions of patients with both elevated RAAS activity and GLU concentrations were determined. Results are shown in Table 3, in which we demonstrated that there was a statistically significantly higher (*P* < 0.05) proportion of patients with a higher GLU in the Ang II elevated group compared with those in the Ang II nonelevated group. 

### 3.4. Multivariate Analysis of GLU Concentration after Treatment

After one year of medication, multivariate analysis was performed using the change of GLU levels as dependent variable against factors that may affect the GLU changes resulting from medication, including gender, age, BMI, baseline GLU level, RAAS changes, and changes in serum K^+^, into the linear regression equation. The results showed that after adjustment for other factors, the serum Ang II levels were independently associated with GLU level after taking HCTZ for one year (Table 4). 

## 4. Discussion

RAAS is one of the main mechanisms through which the body regulates water and salt metabolism. Its activation not only plays an important role in the pathogenesis of hypertension [5], but also can affect insulin resistance. Studies conducted by Scheen [6] have shown that excessive RAAS activity, acting synergistically with microcirculatory changes, can affect pancreas, the major insulin secreting organs, and insulin sensitivity [7] and impair cellular responses to insulin signaling, thereby affecting GLU metabolism. The inhibition of RAAS can increase the adiponectin concentration [8], thus improving B cell function [9] and insulin sensitivity. Studies have also shown that the prevalence of diabetes in hypertensive patients is about 4% to 36% [10], more than in normal patients (3.62%). The prevalence rate of hypertension in patients with impaired glucose tolerance or diabetes was 2 to 3 times that in nondiabetic patients. These facts suggested that a relationship between RAAS activation and glucose metabolism existed and prompted increasing attention drawn to cardiovascular drugs which could affect RAAS activity. As a common diuretic, thiazides can lower blood pressure by reducing blood volume; however, they may also activate RAAS through negative feedback.

Our study showed that there was less reduction in GLU concentrations in patients with elevated Ang II, and the proportions of patients with elevated GLU were higher than those in patients in whom Ang II was not elevated. Multivariate analysis showed that changes in Ang II concentrations were positively correlated with changes in GLU concentrations; that is, there was a statistically significantly smaller decrease in GLU concentrations in patients who had a smaller reduction in Ang II after taking HCTZ for one year (*P* < 0.05). Our results also provided other evidence to confirm that changes in RAAS activity were positively correlated with change in GLU concentration: the higher the RAAS activity, the higher the GLU concentration. These results also suggest that when providing treatment to diabetic patients with hypertension, in order to achieve the desired therapeutic effects, lowering both the GLU concentration and RAAS activity should be considered simultaneously. 

We did not observe a relationship between RAAS activity and blood K^+^ concentrations (data not shown) after administering HCTZ for one year, while previous publications showed that the influence of thiazide diuretics on glucose metabolism was related to reductions in blood K^+^ concentration. We think that the reasons for this inconsistency may include that (1) thiazide diuretics may exert their effects on glucose metabolism through mechanisms unrelated to RAAS activity; (2) effects of diuretics are dosage-related, and the dosage of HCTZ in this study was 12.5 mg daily, a relatively low dosage. This dosage may not have been high enough to influence blood K^+^ concentration; (3) a differential response to HCTZ between the domestic population and patients from other countries could not be ruled out.

We did not observe significant effect on plasma oxLDL level before and after one-year HCTZ treatment. Although the trend indicates a slight reduction of oxLDL, this may be due to the more consciousness of the patients during the treatment towards the more healthy diet.

## 5. Conclusions

There was a correlation between changes in blood Ang II levels and changes in blood GLU concentrations in patients with hypertension after one year of hydrochlorothiazide administration. This result suggests that, when treating patients with hypertension with thiazide diuretics, attention must also be paid to controlling RAAS activity to avoid the negative impact of the drug on GLU metabolism. As there was a one-year follow-up period in this study, longer-term follow-up may be necessary to confirm this conclusion.

## 6. Summary

What is known about this topic is the following.

 (i) RAAS is one of the main mechanisms through which the body regulates water and salt metabolism. Its activation not only plays an important role in the pathogenesis of hypertension [5], but also can affect insulin resistance.
(ii) Excessive RAAS activity, acting synergistically with microcirculatory changes, can affect pancreas, the major insulin secreting organs, and insulin sensitivity [7] and impair cellular responses to insulin signaling, thereby affecting GLU metabolism.

What this study adds is the following.

(i) We found a positive correlation between changes in blood Ang II levels and changes in blood GLU concentrations in patients with hypertension after one year of hydrochlorothiazide administration. When treating patients with hypertension with thiazide diuretics, attention must also be paid to controlling RAAS activity to avoid the negative impact of the drug on GLU metabolism.

## Figures and Tables

**Table 1:** Clinical characteristics of patients after treatment for one year (mean ± sd).

Indexes	Baseline level	Level after one year of medication	*t *	*P *
BUN/mmol/L	5.60 ± 1.67	5.58 ± 1.38	0.111	0.912
Cr/*μ*mol/L	90.68 ± 16.61	91.56 ± 16.51	−0.603	0.548
GLU/mmol/L	6.21 ± 2.37	5.28 ± 2.51	6.053	0
K^+^/mmol/L	4.62 ± 0.60	4.53 ± 0.49	1.398	0.165
TG/mmol/L	1.84 ± 0.10	1.78 ± 0.12	0.543	0.588
TC/mmol/L	6.04 ± 1.08	5.35 ± 1.11	6.537	0
HDL-C/mmol/L	1.49 ± 0.39	1.89 ± 0.72	−6.132	0
LDL-C/mmol/L	3.69 ± 1.09	2.26 ± 0.78	10.783	0
VLDL-C/mmol/L	0.37 ± 0.21	0.41 ± 0.57	−0.895	0.372
oxLDL (RLU)	6591 ± 1592	6075 ± 2223	−1.677	0.187
PRA/ng/mL·h	1.89 ± 1.50	1.46 ± 1.31	2.538	0.013
ACE/U/L	39.60 ± 17.52	41.25 ± 21.81	−0.951	0.344
AngII/pg/mL	67.58 ± 32.17	58.29 ± 44.59	2.482	0.015
ALD/ng/L	152.06 ± 53.14	144.78 ± 68.49	0.802	0.424

**Table 2:** Comparison of GLU concentrations and changes between paired RAAS activity groups after one year of medication (mean ± sd).

RAAS activity after 1 year of medication	Groups	GLU baseline (mmol·L^−1^)	ΔGLU* (mmol·L^−1^)
PRA	Elevated	5.71 ± 3.05	−0.26 ± 0.26
	Nonelevated	5.12 ± 2.44	−1.36 ± 0.23
	*t *	−0.981	−3.502
	*P *	0.329	0.003
ACE	Elevated	5.24 ± 2.39	−0.76 ± 0.23
	Nonelevated	5.29 ± 2.63	−1.09 ± 0.19
	*t *	0.116	−1.083
	*P *	0.908	0.281
Ang II	Elevated	5.58 ± 2.91	−0.17 ± 0.18
	Nonelevated	5.23 ± 2.59	−1.07 ± 0.21
	*t *	−0.576	−2.865
	*P *	0.566	0.005
ALD	Elevated	5.41 ± 2.80	−0.67 ± 0.28
	Nonelevated	5.18 ± 2.44	−1.12 ± 0.19
	*t *	−0.441	−1.338
	*P *	0.66	0.184

*Δ refers to mean changes when compared with baseline level after one year of hydrochlorothiazide.

**Table 3:** Relationship between changes in RAAS and changes in plasma glucose after one year of HCTZ therapy.

RAAS activity		After one year of medication		*χ* ^2^	*P *
		GLU elevated patients	GLU nonelevated patients		
PRA	Elevated group	13	32		
	Nonelevated group	17	58	0.696	0.404
ACE	Elevated group	19	43		
	Nonelevated group	12	45	1.226	0.268
Ang II	Elevated group	17	25		
	Nonelevated group	13	67	8.023	0.005
ALD	Elevated group	16	34		
	Nonelevated group	14	55	2.109	0.146

Data in Table 3 demonstrates that there was a statistically significantly higher (*P* < 0.05) proportion of patients with a higher GLU in the Ang II elevated group compared with the Ang II nonelevated group.

**Table 4:** Multivariate analysis of the change* in plasma GLU level after treatment.

Variable	*β*	SE	*t *	*P *
Gender	0.094	0.346	0.877	0.383
Age	−0.052	0.021	−0.054	0.616
BMI	−0.115	0.05	−1.083	0.282
Baseline GLU	−0.037	0.07	−0.347	0.729
Blood K^+^ change	−0.178	0.23	−1.757	0.083
PRA change	0.079	0.139	0.597	0.552
ACE change	0.181	0.009	1.705	0.092
Ang II change	0.283	0.005	2.616	0.011
ALD change	0.194	0.002	1.744	0.085

*The change of GLU was used as the dependent variable for the analyses.
